# Pediatric diffuse midline glioma H3K27- altered: A complex clinical and biological landscape behind a neatly defined tumor type

**DOI:** 10.3389/fonc.2022.1082062

**Published:** 2023-01-16

**Authors:** Stefano Gabriele Vallero, Luca Bertero, Giovanni Morana, Paola Sciortino, Daniele Bertin, Anna Mussano, Federica Silvia Ricci, Paola Peretta, Franca Fagioli

**Affiliations:** ^1^Pediatric Oncohematology Division, Regina Margherita Children’s Hospital, Azienda Ospedaliera Universitaria (AOU) Città della Salute e della Scienza, Turin, Italy; ^2^Pathology Unit, Department of Medical Sciences, University of Turin, Turin, Italy; ^3^Neuroradiology Unit, Department of Neuroscience, University of Turin, Turin, Italy; ^4^Department of Neuroradiology, Azienda Ospedaliera Universitaria (AOU) Città della Salute e della Scienza, Turin, Italy; ^5^Radiotherapy Unit, Regina Margherita Children’s Hospital, Azienda Ospedaliera Universitaria (AOU) Città della Salute e della Scienza, Turin, Italy; ^6^Child and Adolescent Neuropsychiatry Division, Department of Public Health and Pediatric Sciences, University of Turin, Turin, Italy; ^7^Pediatric Neurosurgery Division, Regina Margherita Children’s Hospital, Azienda Ospedaliera Universitaria (AOU) Città della Salute e della Scienza, Turin, Italy; ^8^Department of Public Health and Pediatrics, University of Turin, Turin, Italy

**Keywords:** H3K27, WHO classification, diffuse midline glioma, pediatric, CNS tumors, brain cancer, pediatric neuro-oncology, WHO CNS 5

## Abstract

The 2021 World Health Organization Classification of Tumors of the Central Nervous System, Fifth Edition (WHO-CNS5), has strengthened the concept of tumor grade as a combination of histologic features and molecular alterations. The WHO-CNS5 tumor type “Diffuse midline glioma, H3K27-altered,” classified within the family of “Pediatric-type diffuse high-grade gliomas,” incarnates an ideally perfect integrated diagnosis in which location, histology, and genetics clearly define a specific tumor entity. It tries to evenly characterize a group of neoplasms that occur primarily in children and midline structures and that have a dismal prognosis. Such a well-defined pathological categorization has strongly influenced the pediatric oncology community, leading to the uniform treatment of most cases of H3K27-altered diffuse midline gliomas (DMG), based on the simplification that the mutation overrides the histological, radiological, and clinical characteristics of such tumors. Indeed, multiple studies have described pediatric H3K27-altered DMG as incurable tumors. However, in biology and clinical practice, exceptions are frequent and complexity is the rule. First of all, H3K27 mutations have also been found in non-diffuse gliomas. On the other hand, a minority of DMGs are H3K27 wild-type but have a similarly poor prognosis. Furthermore, adult-type tumors may rarely occur in children, and differences in prognosis have emerged between adult and pediatric H3K27-altered DMGs. As well, tumor location can determine differences in the outcome: patients with thalamic and spinal DMG have significantly better survival. Finally, other concomitant molecular alterations in H3K27 gliomas have been shown to influence prognosis. So, when such additional mutations are found, which one should we focus on in order to make the correct clinical decision? Our review of the current literature on pediatric diffuse midline H3K27-altered DMG tries to address such questions. Indeed, H3K27 status has become a fundamental supplement to the histological grading of pediatric gliomas; however, it might not be sufficient alone to exhaustively define the complex biological behavior of DMG in children and might not represent an indication for a unique treatment strategy across all patients, irrespective of age, additional molecular alterations, and tumor location.

## 1 Introduction

The integration of genomics into the histopathology of pediatric brain tumors has changed the way we diagnose, classify, and treat brain cancer in children.

In the last two decades, our understanding of the etiology and the biological origin of several types of childhood brain tumors has profoundly improved. Genomics has enriched and supplemented traditional histopathology methodologies: DNA and RNA sequencing, RNA expression profiling, fluorescence *in situ* hybridization, and, finally, DNA methylation have been demonstrated to be valuable tools for refining and improving both the classification and diagnosis of adult and childhood brain cancers. The application of genomic and epigenomic molecular profiling techniques has unveiled a complex biological landscape behind all forms of pediatric brain cancer, revolutionizing our knowledge in the field of pediatric neuro-oncology. We have been moving from a morphology-based to a molecular-based categorization of diseases, in which we now are able to identify many subgroups of tumors characterized by different clinical behavior, prognosis, anatomical location, and age at presentation (1).

The importance of genomics and molecular features of brain tumors started to emerge in the updated fourth edition of the World Health Organization (WHO) Classification of Tumors of the Central Nervous System (CNS) (2016). For the first time, the 2016 WHO CNS classification used molecular parameters in addition to histology to define many tumor entities. It encompassed new sub-classification for diffuse gliomas, medulloblastomas, and other embryonal tumors, and it defined new entities based on their unique molecular features (glioblastoma, IDH-wild-type, and glioblastoma, IDH-mutant; diffuse midline glioma, H3K27M-mutant; RELA fusion-positive ependymoma; medulloblastoma, WNT-activated and medulloblastoma, SHH-activated; and embryonal tumor with multilayered rosettes, C19MC-altered) (2).

The integration of molecular and genomic features in histology then became increasingly important in the fifth edition of the WHO classification of CNS tumors (2021) (3). As far as pediatric CNS tumors are concerned, this led to some peculiar changes in the 2021 classification: (i) there are now “pediatric-type” and “adult-type” tumor families for both low- and high-grade gliomas; (ii) several novel tumor entities of interest in pediatric age have been defined (in many cases, primarily by their molecular characteristics), as shown in **Table 1** (4); (iii) molecular parameters have been integrated into tumor grading, which is a result of combined histological and molecular grading within-tumor-type; (iv) precise molecular diagnostic tools (including in some cases DNA methylation) are indicated as needed for the diagnosis of particular tumor types (3).

**Table 1:** Glioma types of clinical interest in children and adolescents, as per the 2021 WHO Classification of Tumors of the Central Nervous System, Fifth Edition.

Gliomas of clinical interest in children and adolescents	New entity (2021 CNS WHO)	Genetic/molecular alterations
Pediatric-type diffuse low-grade gliomas		
Diffuse astrocytoma, MYB- or MYBL1-altered	x	MYB, MYBL1
Angiocentric glioma		MYB
Polymorphous low-grade neuroepithelial tumor of the young	x	BRAF, FGFR family
Diffuse low-grade glioma, MAPK pathway-altered	x	FGFR1, BRAF
Pediatric-type diffuse high-grade gliomas		
Diffuse midline glioma, H3 K27-altered	refined	H3 K27, TP53, ACVR1, PDGFRA, EGFR, EZHIP
Diffuse hemispheric glioma, H3 G34-mutant	x	H3 G34, TP53, ATRX
Diffuse pediatric-type high-grade glioma, H3-wild-type, and IDH-wild-type	x	IDH-wild-type, H3-wild-type, PDGFRA, MYCN, EGFR
Infant-type hemispheric glioma	x	NTRK, ALK, ROS, MET
Circumscribed astrocytic gliomas		
Pilocytic astrocytoma		KIAA1549-BRAF, BRAF, NF1
High-grade astrocytoma with piloid features	x	BRAF, NF1, ATRX, CDKN2A/B
Pleomorphic xanthoastrocytoma		BRAF, CDKN2A/B
Subependymal giant cell astrocytoma		TSC1, TSC2
Astroblastoma, MN1-altered		MN1

Newly defined entities are marked in the second column. Typical genetic alterations are listed in the third column for each tumor type. Adapted from (3, 4). NB: tumors that are exclusively found in adults, although present in the 2021 WHO CNS classification, are not listed in this table. Glioneuronal tumors and ependymomas, although of pediatric interest, are not listed.

## 2 Pediatric high-grade diffuse midline gliomas, H3K27-altered, and the WHO Classification of central nervous system tumors

Pediatric high-grade gliomas (HGGs), which are among the least curable and most challenging brain neoplasms in children, have been greatly involved in such a crucial biological and histopathological revolution. For many decades, HGGs in children have been considered similar to their adult counterparts. Indeed, in recent years, several genomic studies largely showed that childhood aggressive gliomas are represented by several peculiar biological entities and are not at all the pediatric equivalents of adult malignant gliomas (5). In WHO CNS 2021, pediatric HGGs are formally distinguished from adult HGGs, emphasizing their biological differences. In the pediatric HGG family, four different HGG types are identified: diffuse midline glioma, H3K27-altered; diffuse hemispheric glioma, H3G34-mutant; diffuse pediatric-type high-grade glioma, H3-wild-type, and IDH-wild-type; and infant-type hemispheric glioma (3).

Among pediatric HGG, diffuse midline gliomas (DMG) encompass an apparently homogeneous group of aggressive central nervous system (CNS) neoplasms that can arise in the brainstem (including the formerly defined “diffuse intrinsic pontine gliomas” or DIPG), the thalamus, the cerebellum, the gangliocapsular region, the cerebellar peduncles, the third ventricle, the hypothalamus, the pineal region, and in the spinal cord (6). They represent around 20% of all pediatric CNS tumors, with around 200-300 cases per year in the United States (7). DMGs, and DIPGs in particular, are leading causes of solid tumor death in children; overall, their prognosis has remained extremely poor, and for many years, no significant improvement has been achieved in their treatment (8). The majority of DMGs occur in children aged 5 to 10 years, without any gender predilection (9). Dissemination at diagnosis is possible but rare; secondary metastases are more frequent, being reported in 13% of cases, and can present as intraparenchymal, ventricular, or leptomeningeal (10, 11).

**Figure 1:**
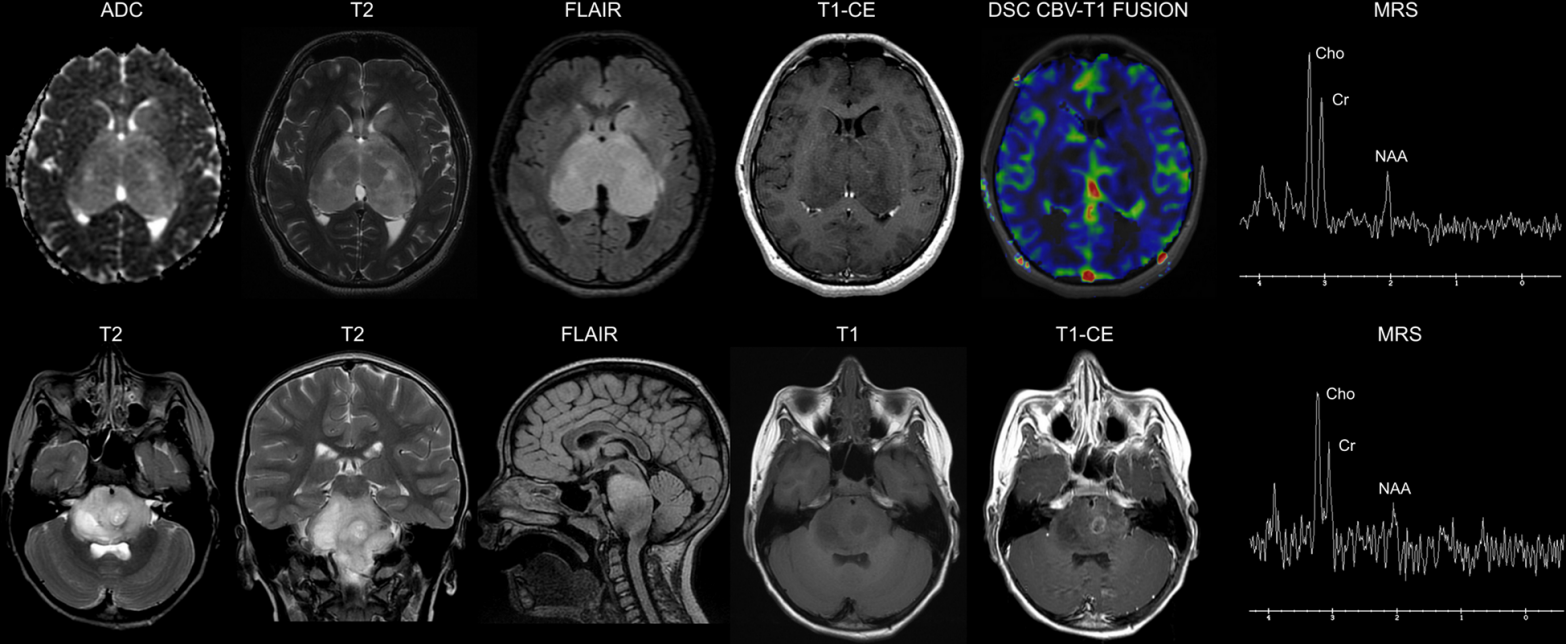
Neuroimaging findings in diffuse midline gliomas H3K27-altered. Upper row: 16-year-old male. Diffuse Midline Glioma, EGFR-mutant. Brain axial Apparent Diffusion Coefficient (ADC) map, T2-weighted, Fluid Attenuated Inversion recovery (FLAIR) and Contrast-Enhanced (CE) T1-weighted images show a bi-thalamic infiltrating and expansile lesion with increased diffusivity and a lack of contrast enhancement. There is concomitant infiltration of the left striatum. Dynamic Susceptibility Contrast (DSC) Cerebral Blood Volume (CBV) perfusion-weighted imaging map fused with T1-weighted imaging shows low perfusion of the lesion. Single voxel Magnetic Resonance Spectroscopy (MRS) with an echo time of 144 ms shows a prominent increase in the Cho/NAA ratio. **Lower row**: 7-year-old male. Diffuse Intrinsic Pontine Glioma (H3.3 K27-mutant). Brain axial and coronal T2-weighted, sagittal FLAIR, and axial T1-weighted images show a diffusely infiltrating lesion involving the pons. The axial CE T1-weighted image shows a left paramedian focal area of ring enhancement. Single-voxel MRS with an echo time of 144 ms shows a marked increase in the Cho/NAA ratio.

Magnetic resonance imaging (MRI) is the gold standard in the diagnosis of DMG and, in particular, of DIPG, in which typical findings include a T1- and T2-hyperintense lesion involving >50% of the pons and high perfusion and restricted diffusion sequences (**Figure 1**) (12–14). Routine biopsy in DIPG remains under debate and is mainly restricted to cases with an atypical imaging appearance (15). A typical DIPG diagnosis may be made based on MRI and clinical criteria only: multiple cranial neuropathies, long tract signs (hyper-reflexia, clonus, increased tone, presence of a Babinski reflex), and ataxia (12). Positron emission tomography (PET) imaging might also find its role as an integrative diagnostic tool in DMG (16).

Current treatment strategies for DMG encompass focal intensity-modulated radiation therapy (IMRT) to the primitive tumor (usually 54–60 Gy in 1.8–2 Gy fractions, given over 6 weeks) and variable lines of chemotherapy. Nonetheless, despite the various attempts at new treatment approaches described so far, the prognosis remains poor (8). Re-irradiation, which represents the only effective treatment for recurrent disease, can lead to symptom relief or neurological improvement in the majority of patients and slightly prolong survival after relapse but remains a palliative and not a curative option (17–21).

Somatic mutations in histone 3 (H3) gene variants H3F3A and HIST1H3B, encoding histone H3 variants H3.3 and H3.1, respectively, collectively referred to as H3K27M (p.Lys27Met), have been detected in the majority of biopsied DIPG and in general in DMG. An H3.2 variant has also been documented (22, 23). The K27M mutant variant causes a global reduction in levels of H3 lysine 27 trimethylation (H3K27me3). In normal cells, trimethylation is mainly established by the H3K27-specific histone methyltransferase enhancer zeste 2 (EZH2) within the Polycomb Repressive Complex 2 (PRC2). Thus, H3K27M results in hypomethylation and ultimately leads to an epigenetic dysregulation of cellular processes due to the inactivation of PRC2, through an interaction between EZH2 and the mutant histone (24, 25).

In the previous 2016 WHO classification, diffuse midline glioma (H3K27M-mutant) was defined as an infiltrative midline high-grade glioma with predominantly astrocytic differentiation and a K27M mutation in either H3F3A or HIST1H3B/C (2).

In 2018, the consortium cIMPACT-NOW (the “Consortium to Inform Molecular and Practical Approaches to CNS Tumor Taxonomy”—Not official WHO), which aims to link the WHO classification effort and the daily work of practicing physicians, clarified one important key-point regarding the diagnosis of “Diffuse Midline Glioma, H3K27M-mutant” (as defined in the WHO 2016 classification), stating that the term Diffuse Midline Glioma, H3K27M–mutant should be used to identify tumors that are diffuse (i.e., infiltrating), midline, gliomas (with the expression of glial markers, particularly Olig2) and H3K27M-mutant, and should not be applied to other tumor types (e.g. non-diffuse gliomas) that are H3K27M-mutant. While at first H3K27M mutations were documented exclusively in DMG, appearing as an exclusive molecular hallmark of such disease, these mutations were later reported in other brain tumors. Nonetheless, the detection of these mutations now seems to confer a strong clinical prognostic value only when they occur in the setting of diffuse midline gliomas (26).

**Figure 2:**
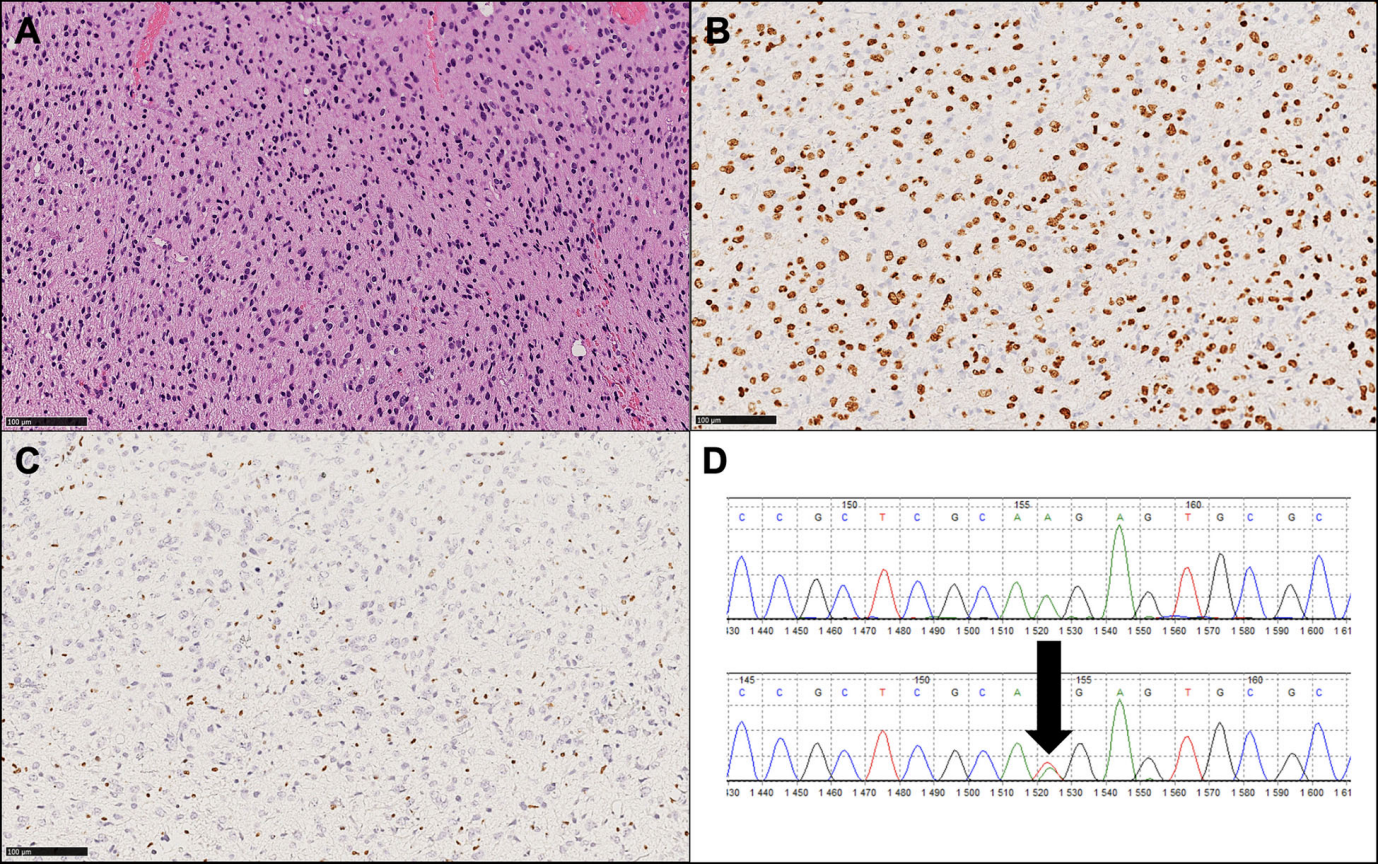
Histopathological and molecular findings of a representative diffuse midline glioma, H3 K27-altered (WHO 2021). **(A)** Hematoxylin and eosin image (original magnification: 100X) showing a diffuse, infiltrative glioma with astrocytic morphology. **(B)** Diffuse expression of OLIG2, a glial marker, is consistent with this tumor type. **(C)** Loss of H3K27me3 is present and exemplifies a mandatory diagnostic feature. **(D)** Sanger sequencing output showing a K27M mutation (arrow), the most frequent molecular alteration observed in this tumor type.

Immunohistochemistry (IHC) is useful for identifying mutations and, in particular, for diagnosing H3K27M-mutant diffuse midline gliomas. IHC is inexpensive, and several studies have reported significant associations between H3K27M protein expression and the H3K27M mutation (27). The assay is widely available nowadays, but its results need to be carefully interpreted; positivity needs to be identified as nuclear staining in neoplastic cells rather than cytoplasmic staining in macrophages and/or microglia. H3K27me3 immunoreactivity is mutually exclusive with H3K27M positivity in most cases, so the loss of H3K27me3 expression should always be analyzed and evidenced in conjunction with H3K27M positive immunohistochemistry (**Figure 2**) (26). It has to be noted that some nomenclatures use the designation K28 rather than K27 to identify the affected lysine residue (28).

H3K27M mutation status can also be assessed by other methods beyond IHC, including Sanger sequencing, next-generation sequencing (NGS), droplet-digital polymerase chain reaction, and pyrosequencing. Indeed, it has been found that DMG can contain sub-clonal, mosaic-pattern H3K27M mutations, and it has been shown that in some cases, tumor cells displayed cytoplasm positivity or lymphocyte immunopositivity and have been later confirmed to be H3 wild-type by Sanger sequencing. Therefore, in some instances, further sequencing is needed to detect the status of H3K27M. It has been shown that IHC can reach almost 100% sensitivity, while Sanger sequencing has 100% specificity. Thus, while IHC is an efficient method for routine use, a combination of IHC and Sanger sequencing (or NGS) is strongly advisable since it can virtually provide 100% sensitivity and specificity for the definition of H3K27M status (29).

Furthermore, it has been recently further demonstrated that in addition to the K27M mutation, other molecular changes can be found in pediatric DMG, namely overexpression of the EZH inhibitory protein (EZHIP) and alterations in the epidermal growth factor receptor (EGFR).

EZHIP overexpression, resulting in H3K27me3 global reduction, has been first observed in posterior fossa type-A ependymomas (30). After having observed that rare cases of DIPG and DMGs lacked a histone H3 mutation, Castel et al. identified nine out of 241 cases (3.7%) displaying a typical infiltrating DIPG histopathology and H3K27me trimethylation loss that, however, lacked K27M positivity by immunohistochemistry (IHC). By analyzing EZHIP expression in DMG, they then identified its systematic overexpression. Importantly, such EZHIP overexpression can be detected by IHC, and Castel et al. ultimately proposed that these EZHIP/H3-WT tumors might be considered similar to K27M mutated DMGs, extending the spectrum of DMG with PRC2 inhibition beyond the H3K27M mutation (31).

More recently, Mondal et al. described the existence of a subset of diffuse gliomas, with mainly thalamic or bithalamic origin that show frequent epidermal growth factor receptor (EGFR) gene amplification and/or mutation and loss of H3K27me3. Loss of trimethylation seems to be mediated by either the H3K27 mutation or EZHIP overexpression (32, 33). The authors concluded that loss of H3K27me3 should then be considered a common feature of three different molecular classes of pediatric DMG: (i) the “typical” DMG with H3K27M mutation, (ii) the DMG with EZHIP overexpression (which additionally shows a high frequency of ACVR1 mutations), and (iii) the mainly bithalamic diffuse gliomas that present H3K27M or EZHIP overexpression together with strong enrichment for EGFR alterations (33).

Taking into account these recent discoveries, which are of paramount biological importance, the 2021 WHO classification of CNS tumors (fifth edition) adopted the revised designation “diffuse midline glioma, H3K27-altered” to include subtypes of DMG with an alternative mechanism for the loss of H3K27 trimethylation (EZHIP overexpression DMG, EGFR mutant DMG), in addition to the most common H3K27M mutation (3). The subclassification of pediatric DMGs according to the 2021 WHO CNS Classification is resumed in **Table 2**.

**Table 2:** Subclassification of pediatric-type diffuse midline gliomas, H3 K27-altered. Adapted from (4).

Diffuse midline glioma, H3.3 K27-mutant	H3.3 pK28M/I (K27M/I) mutation, often co-occurring with TP53/PPM1D mutation and PDGFRA alteration
Diffuse midline glioma, H3.1 or H3.2 K27-mutant	H3.1 or H3.2 pK28M (K27M) mutation, often co-occurring with PIK3CA, PIK3R1 or PTEN mutations, and ACVR1 mutation
Diffuse midline glioma, H3-wild-type with EZHIP overexpression	EZHIP overexpression
Diffuse midline glioma, EGFR- (and H3 K27-) mutant	EGFR mutation (insertion/deletion within exon 20 or p.A289T or p.A289V mutation), often co-occurring with TP53 mutation

## 3 Other tumors with H3K27 mutations

Over the past few years, the same H3K27M mutation has been identified in several tumor types that are not diffuse midline gliomas (26); in particular, it has been reported in ependymomas, pilocytic astrocytomas, pediatric diffuse astrocytomas, and gangliogliomas.

### 3.1 Ependymoma

In 2017, Ryall et al. showed that while K27M mutations can be found, they are extremely rare in posterior fossa type A (PFA) ependymomas, identifying only one case out of 151 harboring the K27M mutation and stating that routine evaluation of K27M mutations in PFA ependymomas is of limited utility and unlikely to have any prognostic role (34). Indeed, more recent studies suggested that PFA ependymomas might be driven by epigenetic changes in DNA and histone methylation, and that while the K27M mutation is actually rare in PFA ependymomas, a global loss of H3K27me3 can be typically observed in PFA ependymomas. Such lower levels of H3K27me3 in PFA ependymomas are due to the overexpression of EZHIP (“enhancer of zeste homolog inhibitory protein”), a protein that might work as a potential tumor driver in PFA and that mimics K27M mutated histones, functioning as an intrinsic inhibitor of PRC2 function (35). Several reports described elevated EZHIP expression in DMG cases that lack H3 mutations, which supports the fact that EZHIP expression and H3K27M mutations are mutually exclusive and are encountered in reverse proportions: 3% versus 97% and 96% versus 4%, respectively, in DMG and PFA ependymomas (30, 31, 36). Interestingly, neither histopathologic distinctions nor outcome differences have been found between PFA EZHIP-overexpressing ependymoma and H3 K27M-mutant ependymoma (37). Nonetheless, investigating further the role of epigenetic changes, loss of H3K27 trimethylation, and EZHIP overexpression in PFA might hopefully lead to a better understanding of the genesis of such tumors and the identification of potential drug targets.

### 3.2 Non diffuse – pediatric low-grade astrocytoma and ganglioglioma

Pilocytic astrocytoma (PA) is the most common brain tumor in children. It is a well-circumscribed tumor with slow growth and is classified as a grade I tumor by the World Health Organization. Malignant transformation (MT) of low-grade gliomas (LGG) is a very unusual event in the pediatric population (38). The H3 K27 mutation in PA is considered to be very unusual, but some reports in the literature tend to suggest a longer survival than K27M DMG. Hochart et al. described the case of a child with spinal pilocytic astrocytoma that had been surgically removed and remained off-therapy without treatment for 10 years. The tumor relapsed 10 years later as a glioblastoma. The exclusive presence of an H3.3- K27M mutation was found in the primary tumor (PA), while both K27M and TP53 mutations were detected in the relapsed tumor (glioblastoma). It might be hypothesized that the H3.3-K27M mutation was the first oncogenic hit, while the TP53 mutation, as the second hit, was responsible for the malignant transformation (39). Jones et al. described a patient with pilocytic astrocytoma and H3.3- K27M in association with somatic NF1 and FGFR1 mutations (40). In a study from 2020, it was shown that patients with H3K27M mutant LGG had significantly lower survival than the wild-type group (median OS, respectively, 17.1 months vs. more than three years), suggesting that in histologically classified LGG, H3K27M mutant tumors should be treated more aggressively (41).

## 4 A complex biological and clinical picture behind a unifying definition

Although the WHO classification offers a clear and net definition of pediatric DMGs (H3K27-altered), many studies have demonstrated some heterogeneity within this unique entity, both from a biological and clinical point of view.

Many different reports seem to indicate that the presence of the H3K27M mutation works as an independent negative prognostic marker in DMGs (22, 23, 34, 41, 42).

Nonetheless, other variables in biology, anatomy, and age could potentially help identify different prognostic sub-categories of DMGs with slightly different clinical behavior, leading to a stratification of patients according to different risk factors.

### 4.1 Biological variables

#### 4.1.1 H3 mutation subtypes

Some authors hypothesized that different subtypes of H3 mutation might impact OS in DMG; in 2015, Castel et al. described differences in clinical behavior according to different subtypes of H3K27M-mutant DMG: HIST1H3B (H3.1) mutant gliomas displayed better prognosis and better response to treatment than H3F3A‐mutant (H3.3) gliomas. It also has been observed that these two groups had different onset ages (younger in H3.1) and locations along the midline (the H3.1 mutation is almost exclusively seen in the brainstem; the H3.3 mutation is more evenly found along the midline) (43). In another study, it has been observed that the H3.3-K27M mutation is present in almost 60–70% of DIPG and is associated with a short OS (median 11 months). The other variants (H3.1 and H3.2) have a relatively longer OS (median 15 months) and a lower risk of metastasis spread (44). Similar results have been described in a study on long-term survivors of DIPG: H3.1-K27M is associated with a longer median OS than H3.3-K27M (45). A very comprehensive systematic review and meta-analysis by Vuong et al. in 2022 included 26 studies with 102 H3.1-mutant DMGs and H3.3-mutant DMGs. H3.1-K27M mutation confers a better prognosis than H3.3-K27M mutation in children, while in the adult population, H3.3-mutated tumors are associated with better survival (46).

#### 4.1.2 Concomitant molecular alterations

The biological picture of DMG has been enriched and made more complex by the finding of several additional molecular alterations that have been described alongside mutations in H3 and beyond the already cited over-expression of EZHIP and alterations or mutations in EGFR. In fact, different authors have shown that DMG exhibits p53 mutations in almost 50% of cases and amplifications or activating mutations of platelet-derived growth factor receptor alpha (PDGFRA) in 35% of cases (47–49). FGFR1 mutations have also been well described, mainly reported in the thalamus, while PDGFRA alterations are more frequent in the pons (50, 51). Mutations of activin receptor type 1A (ACVR1) can also be detected in 21–32% of DMG patients, and they are significantly associated with young age, prolonged survival, and the H3.1 variant (52). Rarer mutations have been reported in a minority of patients: PPM1D, PIK3CA, PIK3R1, PTEN, and ATRX (33, 53, 54). The prognostic meaning of all such additional molecular alterations in DMGs is still largely undefined, although some initial indications have emerged: in a retrospective study of 94 adults and 70 pediatric cases of diffuse midline glioma, age above 18 years (P=0.007), loss of ATRX expression (P=0.032), and Ki-67 index ≤5% (P=0.039) represented independent favorable prognosticators for longer survival across the entire cohort of H3K27M-mutant DMGs (55), while P53 overexpression has been identified as a negative prognostic factor for overall survival by multivariate analysis in another study (56).

#### 4.1.3 BRAF co-mutations

Particular interest has been focused on the presence of BRAF co-mutations: several cases (at least 15 to 20) of H3K27M/BRAFV600E double mutant gliomas have been described in many reports (mainly gangliogliomas, thalamic gliomas, and diffuse supratentorial gliomas), and sometimes such cases showed long survival (42, 51, 57–61). This seems to suggest the presence of a biological overlap between histologically defined low- and high-grade gliomas and may be associated with a better prognosis than expected, compared to BRAF wild-type and H3K27-mutant DMGs.

### 4.2 Anatomical variables: Debulking and tumor location

#### 4.2.1 Debulking

Although the literature is not conclusive, the extent of surgical resection might have prognostic importance in tumors that are at least partially resectable. Karremann et al., in their cohort of 85 pediatric DMGs, observed that survival did not depend on the extent of tumor resection in H3K27M mutated tumors, while it positively influenced the prognosis in H3K27-wild-type midline gliomas with extended resection >90% (42). On the contrary, as far as pediatric thalamic gliomas are concerned, the HERBY Trial results evidenced that in 42 patients with thalamic-based DMG, 28 had DMG *H3K27* mutant tumors, with no differences in outcome compared with other DMGs. However, participants who underwent major debulking or total or near-total resection had longer overall survival (OS): 18.5 months vs. 11.4 months (14). Of note, since H3.1-mutant DMGs are primarily located in the pons and thalamus, the rate of tumor resection for these tumors is lower as compared to H3.3-mutated DMGs (46).

#### 4.2.2 Tumor location

A study by Wang et al. (comprising both children and adults) showed that the H3K27M mutation might have a different prognostic impact based on anatomical location. K27M tumors had a poorer prognosis in infratentorial gliomas compared with the corresponding H3 wild-type tumors (mainly in the brainstem and spinal cord; P <.0001). However, the OS of patients with supratentorial gliomas did not significantly differ between K27M-mutated and H3 wild-type tumors. Furthermore, patients with spinal H3K27M–mutant DMG demonstrated to have a better chance at survival than patients with brainstem DMG (median, 13.2 months vs. 6.6 months), although no statistically significant difference has been recorded. Finally, patients with H3K27M–mutant gliomas in unusual anatomical locations (cerebellum, corpus callosum, lateral ventricle, frontal lobe, and temporal lobe) had a better prognosis compared with those with corresponding tumors in the brainstem (62). Similar results have been recorded by Vuong et al., who tried to stratify patients with H3K27 DMG among more than 800 patients (children and adults). They found that patients with thalamic and spinal cord tumors had significantly better survival than patients with brainstem tumors (46). Furthermore, unlike thalamic tumors, the presence of the H3K27M mutation in DIPG is a much weaker prognostic indicator: wild-type DIPG (approximately 15% of all biopsied cases) has the same unfavorable prognosis as H3K27M-mutant DIPG (63).

### 4.3 Patient’s age as a prognostic variable

Different observations seem to point out that DMGs do not behave the same in children and adults, and in particular that the finding of H3K27 alterations, which has a strong prognostic value in children, is more uncertain as a marker of a worse outcome in adults (when compared to H3 wild-type tumors).

The general characteristics of adult H3K27M-mutant gliomas are very similar to those reported in the pediatric population. As in children, H3K27M mutations are found mainly in midline tumors, suggesting their role as oncogenic alterations in progenitors implicated in the development of midline structures. Nonetheless, location frequency varies between children (in whom H3K27M-mutant gliomas are mainly pontine) and adults (in whom H3K27M tumors seem more frequently located in the thalamus and the spine) (58). In adults and children, these H3K27-mutant DMGs also seem to be associated with a poor prognosis, although no significant difference has been observed between the median survival of H3K27M-mutant and IDH/H3 wild-type gliomas (64). Also, the report by Ebrahimi et al. in 41 DMG (12 pediatric and 29 adult cases) reported that H3K27M mutations are associated with a poorer prognosis in pediatric patients compared to wild-type tumors, while in adult patients these mutations do not significantly influence survival (65). Some authors even documented that in adult patients with DMG, survival may be similar or unexpectedly improved in H3 K27M-mutant tumors compared to wild-type midline gliomas (66).

On the opposite side of the epidemiological spectrum of age, it has been reported that very young children with DMG (less than three years old) might have a significantly longer OS than older patients (42, 67).

## 5 Discussion

In the medical process of diagnosing and treating patients with cancer, the roles of the pathologist and the physician are often considered to be distant.

Pathologists are primarily committed to obtaining a diagnosis with adequate timing and maximal accuracy to correctly identify the pathology and its sub-types. Moreover, pathologists have a particular interest in disclosing the biological and molecular features of each tumor, since these alterations play a crucial role in the categorization of a disease.

Physicians, on the other hand, are primarily focused on treating patients successfully by administering effective therapies in a timely manner and avoiding toxic or unnecessary treatments. As oncologists, they are interested in the biological and molecular characteristics of the tumor, which can help define the best therapeutic strategy, especially in a modern setting of targeted and personalized medicine.

Any nosological classifications of diseases should be conceived and refined to guide pathologists in making correct and precise diagnoses with a high concordance rate and a low risk of diagnostic error. At the same time, classifications must function as practical tools for clinicians: the histological types and sub-types should, where possible, correlate with the clinical behavior of the disease, the prognosis, and the age of patients to guide physicians in providing the most appropriate therapies.

The 2021 WHO Classification of CNS Tumors (fifth edition) achieved the goal of being a precise and detailed descriptive categorization of diseases while also providing indications with strong clinical and practical value in many ways. This ambitious goal has been pursued in a variety of ways: terminological simplification, often aimed at avoiding misunderstandings (as in the case of the grade written in Arabic rather than Roman numerals); the separation of some pediatric tumors from those of adults (to avoid clinicians being forced to deal with the sub-classifications of entities that they never meet in their clinical practice); and the integrated use of molecular biology and genetics in the definition of nosological entities, more than the mere evaluation of the morphological characteristics of tumors (26).

**Figure 3:**
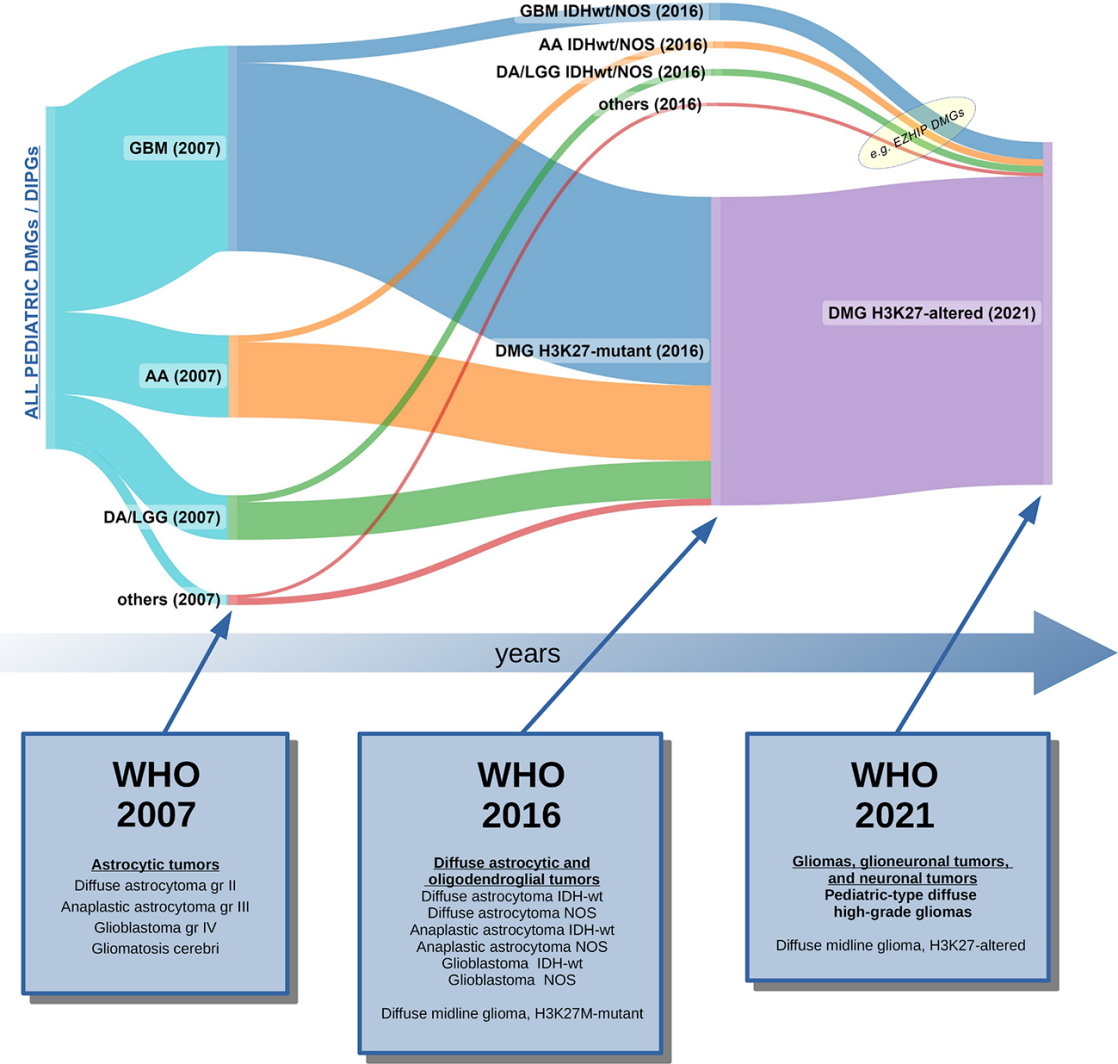
Graphic representation of how pediatric DIPGs and DMGs have been classified over the last 15 years, based on the current and the past WHO Classifications of CNS tumors (2, 3, 68). In 2007, DIPG/DMG was not recognized as a distinct entity: data from autopsies and rare biopsies showed that DIPG/DMGs were histologically classifiable as glioblastomas (GBM gr. IV), or less frequently, anaplastic astrocytomas (AA gr. III), low-grade gliomas (mainly diffuse astrocytomas, LGG-DA gr. II), or other rarer histotypes (69). In 2016, the majority of cases were classified as DMG, H3K27M-mutant tumors, although some non-K27M-mutant DMG still remained unclassified. The 2021 WHO CNS Classification unified all cases of pediatric DMGs in which a H3K27 alteration was found (loss of H3 K27me3 trimethylation). Legend: GBM, glioblastoma; AA, anaplastic astrocytoma; LGG, low-grade glioma; DA; diffuse astrocytoma; IDHwt, IDH wild-type; NOS, not otherwise specified; DMG, diffuse midline glioma; DIPG, diffuse intrinsic pontine glioma.

The WHO classification has been updated and revised in just a few years (between 2016 and 2021) by accepting and integrating the suggestions of cIMPACT-NOW (a consortium created to bridge the gap between the categorization needs and the clinical contextualization of diseases) (26) into the newer version. As far as the main object of this review is concerned, it is important to note that the 2021 classification, taking into account the observations of cIMPACT-NOW, has changed the name of the entire category of tumors: from the original name of 2016 (“diffuse midline glioma, H3K27M-mutant”), it has changed in 2021 to the more generic term “diffuse midline glioma, H3K27-altered,” in order to include the new biologic sub-variants that have been described between 2018 and 2021 (EZHIP and EGFR variants) (**Figure 3**) (3).

The newly named tumor type “Diffuse midline glioma, H3K27-altered” (within the “Pediatric-type diffuse high-grade glioma” family) tries to ideally represent a perfect integrated and homogeneous diagnostic entity in which age (pediatric), location (midline), histology (diffuse glioma), and genetics (H3K27 alteration) are clearly defined, apparently identifying unequivocally a precise and definite tumor entity.

Such a well-defined pathological categorization has a significant impact on the entire pediatric neuro-oncology community. Clinicians usually value precisely classified and clearly defined nosologic entities, especially when they identify diseases that are characterized by a homogeneous prognosis and univocal treatment. However, the identification of the H3K27 alteration in a case of DMG carries in and of itself a very high risk of simplification in everyday clinical practice. In fact, it might in many cases override the histological, radiological, and clinical peculiarities of each individual patient. For a pathologist, the detection of any H3K27 alteration in the presence of a DMG overrides the need for grading the tumor, which has previously been a heavy responsibility for pathologists because incorrect grading could result in a radical change in the treatment strategy (for example, labeling a pediatric diffuse glioma as “high-grade” would have paved the way for radiotherapy, whereas labeling it “low-grade” would have maybe authorized an initial watch-and-wait strategy). Nonetheless, clinicians are now warned to consider all H3K27-altered diffuse midline gliomas as malignant, incurable diseases, and thus they are inclined to treat aggressively all patients with such a diagnosis, regardless of age, duration of symptoms, neurological deficits at presentation, tumor location, and the presence of concomitant mutations beyond H3K27.

We have been learning that all tumors labeled as H3K27-altered DMG globally share the same dismal prognosis: they are aggressive gliomas, not amenable to radical surgery, that respond only to radiotherapy and just for a limited period of time, and then progress lethally in more than 90% of patients within one year (8). Various medical approaches using neoadjuvant or post-irradiation chemotherapy have been tested over the decades, but none has demonstrated that it is able to substantially improve OS or PFS, sometimes resulting in increased toxicities and the need for hospitalization (70–72).

However, is it truly this straightforward? Should we really treat each patient with an H3K27-altered DMG in the same manner?

In actuality, the diagnosis of H3K27-altered DMG will easily lead physicians to communicate the same dismal prognosis to all patients with such a diagnosis, regardless of their age, the location of their tumor, or the presence of other concomitant gene mutations or alterations. Most likely, each patient will be given front-line radiotherapy in the hopes of having the longest possible post-radiotherapy free-of-symptoms honeymoon, after which they will be either enrolled in some promising early-phase clinical trial or receive metronomic chemotherapy and/or palliative re-irradiation.

We do agree with the core of the mainstream message: H3K27-altered DMG are almost invariably aggressive tumors for which there is no effective treatment other than palliative radiotherapy.

Nonetheless, this last sentence contains the two concepts that we would like to primarily emphasize in our review: (i) first, H3K27-altered DMGs are “*almost”* always aggressive tumors, but there are very few cases in which, unexpectedly, some patients have long survival; (ii) second, H3K27-altered DMG “*currently”* have no effective treatment, but we believe that restless investigations by clinicians and biologists will likely soon change again the way we classify these tumors, and hopefully the way we learn to treat and cure them.

### 5.1 DMG are almost invariably aggressive tumors

H3K27 mutations seem to have a strong prognostic value only in diffuse midline gliomas, while in non-diffuse gliomas, non-midline gliomas, or tumors that are not gliomas, their clinical importance is much lower. In fact, the detection of H3K27 alterations in pilocytic glioma, ganglioglioma, and ependymoma does not have the same strong role in characterizing the tumor’s malignancy and biological and clinical behavior as it does in DMGs. It is then very hard to determine to what extent the malignant potential of DMGs is attributable to their intrinsic location (and thus their inoperability and scarce druggability), to their diffuse nature (and so their propensity to infiltrate the normal surrounding brain tissue), or to the biological aggressiveness given by the H3K27 mutation (23).

The prognosis of H3K27 DMG is not identically and homogenously dismal across ages; very young children with DIPG/DMG fare better, and in adult patients, the role of H3K27M as a prognostic indicator is far from being clear and definite. Thus, as pediatric oncologists, when we talk to parents of a child with DMG, perhaps we should modify our communication about prognosis and life expectancy in very young children (because they can have longer than usual survival) or late adolescents (because their tumors may occasionally resemble more those of adults rather than children). Furthermore, we should be aware that in such cases, rare but precious examples of unexpectedly long survival can happen, so we should focus our clinical skills on eagerly trying to transform our patient into one of these fortunate outliers.

Another factor that seems to influence survival is tumor location: while typical intrinsic pontine DMGs are inoperable and in most cases rapidly progress after radiotherapy, other tumors are amenable to partial surgery (partially exophytic tumors, thalamic, cerebellar, and spinal tumors, for example). Although the impact of partial resection in children with H3K27-altered tumors is not as clear as in adults with malignant glial tumors, surgery might sensibly improve prognosis, especially in patients with thalamic and spinal DMGs.

The presence of rare long-term survivors among patients with H3K27-altered DMG (and formerly with DIPG or other midline tumors) has been reported in the literature, both in single case reports and in population studies. In a study of over a thousand DIPG cases by Hoffmann et al., approximately 10% of the patients survived more than two years after diagnosis. Such long-surviving patients more commonly presented at ages <3 or >10 years; they had longer symptom duration and less commonly presented with cranial nerve palsy, ring enhancement, necrosis, and extra-pontine extension; the HIST1H3B mutation also seemed more likely to be found in long-term survivors (45). Indeed, such observations, although representing a major and valuable contribution to the research field of DMG, must be cautiously interpreted, as pointed out in a specific commentary (73): the possibility of enrollment bias or variations in the standard of care between countries and institutions might influence the interpretation of results.

Even more interesting is the presence, in certain reports, of very long-term survivors of DIPG and DMG (e.g., patients surviving more than five years from diagnosis). Such patients, in the current setting, are to be considered real “outliers,” and are reported to account for around 2.5-6.9% of all patients with DIPG (45, 74, 75). Although H3K27 alterations do define this category of DMGs in children, the role of the pathologist must not be minimized as being the person who just writes down a diagnosis that is disclosed by the H3 analysis. Assessment of H3 mutation status alone, especially by the use of IHC alone, is not sufficient to distinguish the more frequent “typical” H3K27M-mutant DMG from the EZHIP or EGFR-altered DMG. Moreover, many additional molecular alterations (PDGFR, ATRX, P53, ACVR1, BRAF, and many others) may be found in DMG alongside H3 mutations that can refine the diagnosis and sometimes change the prognosis. Furthermore, defining the subcategory of histone mutation (H3.1 vs. H3.3) can be of clinical interest since it has been demonstrated to have an influence on survival.

The global biological picture has been made even more complex by the recent demonstration that DMGs are characterized not only by a wide range of inter-tumoral genetic variability but also by a relevant intra-tumoral genomic heterogeneity, with the coexistence of genetically distinct subclones in each tumor, as seen by whole genome and exome sequencing (76, 77). Other techniques, such as single-cell mass cytometry, yielded similar results, revealing significant inter- and intra-tumoral heterogeneity at the protein level (78). H3K27-altered DMG is probably made of multiple, genotypically, and phenotypically distinct subpopulations of tumor cells: this may result in resistance to therapy and exacerbate clinical malignancy. Differences have also been detected across multiple tumor samples collected throughout the brain at autopsy, revealing branching evolutionary trajectories within the same tumor. In rare cases, researchers found distinct low-grade and high-grade components in the same tumor specimen, with key oncogenic mutations in one region but not the other (76).

Indeed, the complete molecular characterization of each DMG case would be of paramount importance for the individual patient and future patients. Although at present this might have limited clinical relevance, a complete molecular characterization of each case of DMG may be critically important in the future, especially if relevant and durable responses to targeted therapeutic approaches are evidenced (33). Such molecular alterations become even more important when coupled with non-uniform histological features: as an example, they can be ancillary in guiding decisions if a tumor shows morphological aspects of diffuse glioma together with features of a glioneuronal tumor. Cases presenting H3K27 alterations that are either “not-so-diffuse”, “not-so-midline” or “not-so-glioma” tumors should be thoroughly examined from a molecular point of view (DNA and RNA NGS, and methylation if possible) in search of additional alterations (e.g., BRAF V600 mutations); furthermore, in such cases, a centralized pathological revision or second opinion is always needed before attributing a definitive category to the tumor.

### 5.2 H3K27-altered DMG currently has no effective treatment

Despite all past and present efforts to find a cure for DIPG and malignant DMG, the vast majority of patients with H3K27-altered DMG will not survive the disease.

This is why conventional treatment for such tumors (currently encompassing radiotherapy and variable subsequent schemes of low-dose medical therapy with little or no impact on survival) is sometimes thought of as a front-line palliative approach. In such a setting, one of the most important aspects of the care of children and adolescents with DMG is avoiding unnecessary toxic treatments, useless hospitalization, and invasive diagnostic procedures (17).

The possibility of using MRI as a widely accepted gold standard for diagnosis in DIPG, in conjunction with the risk of performing a biopsy on intrinsic pontine lesions, has sparked a long and unresolved debate over the need for performing a biopsy in DIPGs versus treating patients on the basis of a radiological diagnosis alone over the last decades. A tumor biopsy is not required for DIPG diagnosis and is only unavoidable in cases of atypical radiological features, although sometimes it is a mandatory requirement for inclusion in a clinical trial (48, 79–81).

Numerous clinical trials have been testing new therapeutic approaches with DMG-targeted drugs. Candidate drugs and compounds include monoclonal antibodies, small molecules, tyrosine kinase inhibitors, angiogenesis inhibitors, and more. Some recent non-intensive approaches suggest slight advantages over the standard of care (frontline radiotherapy alone): there are some encouraging reports on the use of nimotuzumab, a humanized anti-EGFR antibody, with similar outcomes to more intensive chemotherapy regimens, with a lower burden of toxicity and no need for prolonged hospitalization; its use is described in particular in combination with vinorelbine and radiation and re-irradiation by the Milan group (82). The use of personalized, biopsy-based targeted therapies has been investigated, and in some reports, it seemed to produce a slight improvement in prognosis, and low toxicity (81). The use of adoptive T cell therapy is also a promising approach that has been recently tested preclinically and clinically in the context of DMG: a few clinical trials are currently recruiting patients for the use of CAR-T cells in DIPG, and the very first results are encouraging (83–85). Moreover, the use of intratumoral infusion of oncolytic viruses followed by radiotherapy has also been reported (86). The description of the rationale and results of such new therapeutic approaches is beyond the scope of this review. That said, so far, none of the recent clinical trials with published results has demonstrated a relevant impact on improving survival (8), although many of them are still ongoing and many others are not active yet. Much hope and scientific effort are being invested in those modern approaches. Such strategies find their theoretical foundation in the identification of one or more molecular abnormalities in the tumor tissue. As such, these approaches support and motivate a biopsy assessment of the tumor to discover potential therapeutic targets. Ethical concerns about the decision to biopsy all patients are still legitimate, especially in centers where a biopsy is not routinely performed outside the setting of a clinical trial. Nonetheless, the role of biopsy has been gradually reconsidered in recent years, as it is the only way for biology-driven translational research to lead us to an understanding of the mechanisms underlying DMGs and the possible development of more promising clinical trial studies and targeted therapies. Thanks to the development of modern surgical techniques, the procedural risk of biopsies in DIPG has lowered over time, and many report biopsy as a relatively safe technique in experienced centers (60, 79, 87, 88).

In the general setting of pediatric DMGs, independent of their localization, the possibility to obtain extensive information on each and every tumor is of paramount importance in collecting the maximum amount of information about this aggressive disease. The molecular characterization of DMGs may reveal itself to be important not only for the broader aim of determining future treatment strategies but also for the treatment of individual patients. In fact, several studies reported that some patients have benefited from molecularly driven personalized therapies based on the extensive genomic analysis of their tumors (60, 68, 81, 87).

## 6 Conclusion

The WHO classification of central nervous system tumors has rapidly evolved over the last few years. In 2007, diffuse intrinsic gliomas of childhood were not even cited as a separate entity (69); by 2021, the classification had completely changed, separating pediatric HGG and LGG from other gliomas of adulthood and defining the tumor type as “diffuse midline glioma, H3K27-altered” (3). It is therefore plausible to predict that within a few years there may again be some changes in the classification, which would redefine the way we categorize DMG and malignant gliomas in children. Most likely, new discoveries and research will also provide additional information on the significance of histone mutations in gliomas, perhaps to the point of changing their biological significance and prognostic role.

When interpreting nosological classifications of diseases, it’s important to be aware that these are based on current knowledge and scientific discoveries, which are constantly changing and being updated. In clinical practice, as pediatric pathologists, and oncologists, we must therefore act on a case-by-case basis, adapting the indications that the WHO 2021 CNS classification provides and taking into account the clinical and demographic characteristics of every single patient, together with the radiological, histological, biological, and molecular characteristics of their tumor.

Especially when we take care of patients with diseases that are universally characterized by poor survival and few curative therapies, such as H3K27-altered DMG, we must always be alert to identify exceptions, and we must almost spasmodically search for “outliers” among our patients to tailor a specific treatment to them and offer an otherwise minimal chance of cure.

It is our duty to capitalize on the strengths and innovative, modern information offered by the 2021 WHO CNS classification in the management of pediatric patients with H3K27-altered DMG. Nonetheless, we must give each DMG case its own unique and precise molecular characterization. The ultimate goal is to treat all patients with a personalized therapy tailored to the specific characteristics of their tumor, if possible within a clinical trial of molecular medicine, in order to obtain innovative hope for a cure despite the presence of the H3K27 alteration that characterizes the poor prognosis of pediatric DMGs.

## Author contributions

All authors listed have made a substantial, direct, and intellectual contribution to the work, and approved it for publication.
